# A novel multiplex PCR-RFLP method for simultaneous detection of the *MTHFR 677 C > T*, *eNOS +894 G > T* and *- eNOS -786 T > C* variants among Malaysian Malays

**DOI:** 10.1186/1471-2350-13-34

**Published:** 2012-05-17

**Authors:** Keat Wei Loo, Lyn Robyn Griffiths, Siew Hua Gan

**Affiliations:** 1 Human Genome Centre, School of Medical Sciences, Universiti Sains Malaysia, 16150 Kubang Kerian, Kelantan, Malaysia; 2 Genomics Research Centre, Griffith Health Institute, Griffith University, Parklands Drive, Southport, Queensland, Australia

## Abstract

**Background:**

Hyperhomocysteinemia as a consequence of the MTHFR 677 C > T variant is associated with cardiovascular disease and stroke. Another factor that can potentially contribute to these disorders is a depleted nitric oxide level, which can be due to the presence of *eNOS* +894 G > T and *eNOS −*786 T > C variants that make an individual more susceptible to endothelial dysfunction. A number of genotyping methods have been developed to investigate these variants. However, simultaneous detection methods using polymerase chain reaction-restriction fragment length polymorphism (PCR-RFLP) analysis are still lacking. In this study, a novel multiplex PCR-RFLP method for the simultaneous detection of MTHFR 677 C > T and *eNOS* +894 G > T and *eNOS −*786 T > C variants was developed. A total of 114 healthy Malay subjects were recruited. The MTHFR 677 C > T and *eNOS* +894 G > T and *eNOS −*786 T > C variants were genotyped using the novel multiplex PCR-RFLP and confirmed by DNA sequencing as well as snpBLAST. Allele frequencies of MTHFR 677 C > T and *eNOS* +894 G > T and *eNOS −*786 T > C were calculated using the Hardy Weinberg equation.

**Methods:**

The 114 healthy volunteers were recruited for this study, and their DNA was extracted. Primer pair was designed using Primer 3 Software version 0.4.0 and validated against the BLAST database. The primer specificity, functionality and annealing temperature were tested using uniplex PCR methods that were later combined into a single multiplex PCR. Restriction Fragment Length Polymorphism (RFLP) was performed in three separate tubes followed by agarose gel electrophoresis. PCR product residual was purified and sent for DNA sequencing.

**Results:**

The allele frequencies for MTHFR 677 C > T were 0.89 (C allele) and 0.11 (T allele); for *eNOS* +894 G > T, the allele frequencies were 0.58 (G allele) and 0.43 (T allele); and for *eNOS −*786 T > C, the allele frequencies were 0.87 (T allele) and 0.13 (C allele).

**Conclusions:**

Our PCR-RFLP method is a simple, cost-effective and time-saving method. It can be used to successfully genotype subjects for the *MTHFR 677 C > T* and *eNOS +894 G > T* and *eNOS −786 T > C* variants simultaneously with 100% concordance from DNA sequencing data. This method can be routinely used for rapid investigation of the *MTHFR 677 C > T* and *eNOS +894 G > T* and *eNOS −786 T > C* variants.

## Background

Methylenetetrahydrofolate reductase (MTHFR, EC 1.5.1.20) is a flavin adenine dinucleotide (FAD)-dependent enzyme that irreversibly catalyzes the conversion of 5, 10-methylenetetrahydrofolate into 5-methyltetrahyrofolate. The potent methyl donor 5-methyltetrahyrofolate enters a remethylation homocysteine pathway to produce the amino acid methionine [1], which in turn ensures a continuous biochemical pathway that maintains homocysteine at healthy levels.

The accumulation of homocysteine has been shown to be associated with carotid plaque thickness and carotid stenosis, which have vital roles in clot formation and can contribute to cardiovascular disease as well as stroke. [2]. Hyperhomocysteinemia is a consequence of single nucleotide polymorphisms (SNPs) in *MTHFR 677 C > T* that can cause homocysteine levels in the blood to increase, usually exceeding 15 μmol/L. *MTHFR 677 C > T* is the result of a missense mutation occurring at position 222, where alanine is substituted with valine. The mutation results in a thermolabile MTHFR that has lower enzyme activity at 37°C and alters flavin adenine dinucleotide (FAD) dissociation kinetics, leading to increased total plasma homocysteine [3].

Hyperhomocysteinemia predisposes a person to endothelial vascular injury because it can lead to loss of important endothelial vasodilator properties that are regulated by nitric oxide (NO) and endothelial nitric oxide synthase (eNOS). The condition is exacerbated when genetic variation present in *eNOS* leads to a further reduction in NO production. Previous studies have reported that *eNOS +894 G > T* and *eNOS −786 T > C* are associated with silent brain infarction and adverse cerebrovascular events [4].

Barbaux et al. [5] developed a multiplex heteroduplexing method to genotype SNPs for MTHFR and methionine synthase (MS). Although this method has the potential for generating reliable, high-quality data and is cheaper when compared to a real-time PCR method, it is complex due to the necessity of performing an additional cloning step for generating the heteroduplexes. The analytical time is also lengthy, requiring at least 16 hours for genotyping of only a single SNP. Moreover, their method utilized a polyacrylamide gel at a high concentration (12%) for separation, which may pose a health hazard to the researchers.

Another study performed simultaneous detection of *MTHFR 677 C > T* and *1298 A > C* variants using a simple polymerase chain reaction-restriction fragment length polymorphism (PCR-RFLP) method, the first multiplex PCR method reported for *MTHFR*[6]. However, the study has some limitations due to the fact that the enzyme (MboII) was unable to recognize specific sequences at the SNP site; it is more specific for 5′-GAAGA(N)_8_-3′, which results in the formation of non-specific bands. Furthermore, the study was only validated using uniplex PCR-RFLP with no positive controls. DNA sequencing validation was also not performed.

Koksal et al. [7] developed a fast primer-engineered multiplex PCR-RFLP method for the detection of *MTHFR 677 C > T*, prothrombin G20210A and factor V Leiden. The advantage of their method is the use of a single restriction enzyme (MnlI) for simultaneous detection. However, the method required the use of expensive equipment such as an icycler® (Bio-Rad), and the authors also reported that their method is not applicable to routine work that involves the analysis of a large number of samples.

Agarwal et al. [8] used a more advanced real-time multiplex PCR method for the simultaneous detection of *MTHFR 677 C > T* and *1298 A > C* that moved away from conventional PCR methods. With improved probe design, the researchers were able to detect melting peaks with higher resolution, thus reducing the error in interpreting RFLP results. This overcomes the disadvantages of using a conventional PCR-RFLP method. However, the use of this advanced technology is still too costly for routine applications.

Using a conventional PCR-RFLP method, Hayati et al. [9] genotyped 20 healthy Malays as controls and 22 patients with neural tube defects. Overall, 92.9% had the homozygous CC genotype, 7.1% had the CT heterozygous genotype and no homozygous variants were reported. They concluded that there is no association between the *MTHFR 677 C > T* polymorphism and neural tube defects. However, their study is limited by the small sample size.

In another study, Ghazali et al. [10] investigated the frequency of the *eNOS +894 G > T* polymorphism in 200 hypertensive Malays and 198 age- and sex-matched controls and reported the overall genotype frequencies to be 74% (GG homozygote), 25% (GT heterozygote) and 1% (TT homozygote). They performed a conventional PCR-RFLP method using the enzyme MboI, which can be time-consuming. Furthermore, the researchers only genotyped a single SNP of *eNOS.*

The *eNOS −786 T > C* polymorphism has been shown to be associated with silent brain infarction [4]. In Malaysia, six new stroke cases occur every hour [11]. To our knowledge, this polymorphism has not been investigated among Malaysian Malays; for further understanding of the pathogenesis of stroke, this population should be investigated. Therefore, there is a need to develop a simultaneous detection method that is simple, robust, fast and inexpensive for genotyping risk markers for stroke among Malays. We aim to undertake this task by developing a novel multiplex PCR-RFLP method for the simultaneous detection of *MTHFR 677 C > T* and *eNOS +894 G > T* and *eNOS −786 T > C* gene polymorphisms.

## Methods

### Overview

The multiplex PCR was developed in accordance with the Qiagen® Multiplex PCR Handbook [12]. Primer 3 Software (version 0.40) was used to design specific and functional primers for the regions of interest. The primer specificity, functionality and annealing temperature were tested using uniplex PCR methods that were later combined into a single multiplex PCR. As a standard procedure prior to PCR, the DNA concentration and purity were determined using Infinite® 200 NanoQuant (Tecan, Switzerland). Good-quality DNA was indicated by a 260 nm / 280 nm absorbance ratio between 1.8 and 2.0 [13].

### Sample Collection

This was a prospective study conducted at the Hospital Universiti Sains Malaysia (HUSM) between October 2010 and January 2011. This study has been approved by the local institutional review board at the Universiti Sains Malaysia and complies with the declaration of Helsinki.

All Malays confirmed by three generations were healthy subjects (n = 114) and comprised staff and students of the Universiti Sains Malaysia between the ages of 18 and 55 years. They were screened using the inclusion and exclusion criteria before obtaining written informed consent. These subjects were randomly selected healthy volunteers with no previous history or family history of stroke, ischemic heart disease or migraine. Subjects who took B-vitamin complex supplements for a year were excluded from the study.

### DNA extraction

Blood (1 mL) was withdrawn from the subjects and stored in EDTA tubes (Fluka, USA). Genomic DNA was extracted from whole blood (200 μL) using the QIAamp® Blood Mini Kit (Qiagen, Hilden, Germany) with a Spin Protocol before genotyping.

### PCR primers

The primers used for the analysis of *MTHFR 677 C > T*, *eNOS +894 G > T* and −*786 T > C* SNPs using multiplex PCR are described in Table 1. The 677 F and 677R primers were designed with Primer 3 Software version 0.4.0 for the analysis of *MTHFR 677 C > T*[14]. Prior to use, the selected primer pair was validated against the BLAST database [15] to check for primer specificity [7] and eliminate any possibility of repetitive sequences. Further hypothetical sequences generated from the primers were retrieved from the UCSC Genome Bioinformatics server [16], while hypothetical lengths of RFLP products were determined using Restriction Mapper version 3 [17].

**Table 1:** **Primers for *MTHFR 677 C > T*, *eNOS +894 G > T *and *eNOS -786 T > C***

**Primer**	**Sequences**	**Length (bases)**	Tm* (°C)	**GC (%)**	**References**
677 F	5′-CTC GCC TTG AAC AGG TGG AG-3′	20	58.3	60	Self-designed
677R	5′-CTG GAT GGG AAA GAT CCC GG-3′	20	58	60	Self-designed
894 F	5′-GTC CCT GAG GAG GGC ATG AG-3′	20	59.9	65	[18]
894R	5′-TCC AGC AGC ATG TTG GAC AC-3′	20	58.2	55	[18]
786 F	5′-GCA GGT CAG CAG AGA GAC TA-3′	20	55.9	55	[19]
786R	5′-GAC ACA GAA CTA CAA ACC CC-3′	20	53.6	50	[19]

*Tm = melting temperature of primers.

### Multiplex Genotyping

A total PCR reaction mixture of 40 μL was prepared on ice. The mixture consisted of double distilled water, 1 X PCR Buffer II (100 mM Tris–HCl, pH 8.3, 500 mM KCl) (ABI, USA), 1.5 mM magnesium chloride (ABI, USA), 600 μM GeneAmp® dNTP Blend (ABI, USA), 0.5 pmol of each forward and reverse primer (677 F, 677R, 894 F, 894R) (NHK Bioscience Solution® Oligonucleotide, Korea), 0.6 pmol of the 786 F and 786R primers (NHK Bioscience Solution® Oligonucleotide, Korea), 2.0 U of AmpliTaq Gold® DNA polymerase (ABI, USA) and 50–100 ng of the DNA sample. A negative control containing doubly distilled water instead of genomic DNA was also prepared. Samples previously sequenced and found to contain mutations were used as positive controls. The reaction mixture was gently tapped and centrifuged before each PCR.

PCR parameters consisted of an initial denaturation step for 4 min at 95°C, followed by 35 cycles of 30 sec at 95°C, 1 min at 61.1°C and 1 min 30 sec at 72°C and a 7 min final extension step at 72°C.

### Endonuclease Restriction Assay – RFLP method

Before the RFLP, the PCR products were separated into three tubes for the analysis of the *MTHFR 677 C > T*, *eNOS +894 G >* T and *eNOS −786 T > C* variants.

The first and second tubes were used to investigate the *MTHFR 677 C > T* and *eNOS −786 T > C* variants. In these tubes, the RFLP mixture consisted of 8.5 μL of doubly distilled water, 0.1-0.5 ng of fresh PCR product, 1 X Fermentas Fast Digest® Buffer (Fermentas, Lithuania), 1 U Fermentas Fast Digest® HinfI restriction enzyme (Fermentas, Lithuania) and 1 U Fermentas Fast Digest® MspI restriction enzyme (Fermentas, Lithuania). The RFLP reaction mixture was gently tapped and briefly centrifuged before processing.

The third tube was used for the analysis of *eNOS +894 G > T*. For this, 3.9 μL of doubly distilled water, 0.1-0.5 ng of fresh PCR product, 1 X NEBuffer 4® (20 mM Tris-acetate, 50 mM potassium acetate, 10 mM magnesium acetate, 1 mM Dithiothreitol, pH 7.9) (NEB®, England) and 1 U of NEB® BanII restriction enzyme (NEB®, England) were added to a PCR tube.

All three tubes were then incubated at 37°C for 30 min using an Accublock Digital Dry Bath (Alpha Innotech, USA) before PCR analysis.

### Agarose gel electrophoresis

High-resolution agarose gels (5%) (Bioline®, USA) were prepared and immersed into an electrophoresis gel chamber containing 1 X TAE buffer (Fluka, USA). Fermentas 6 x Orange DNA Loading Dye® (1 μL) (Fermentas, Lithuania) was mixed with 1 μL of 100 X GelStar® (Lonza, USA) and 1 μL of Fermentas O’ GeneRuler® Ultra Low Range DNA Ladder [this consisted of 10 mM Tris–HCl (pH 7.6), 10 mM EDTA, 0.025% orange G, 0.005% xylene cyanol FF and 10% glycerol (Fermentas, Lithuania)] or DNA sample on Parafilm® (Parafilm, USA) before adding into the well.

For PCR and PCR-RFLP products, 4 μL of each product was mixed with 1 μL of Fermentas 6 x Orange DNA Loading Dye® [10 mM Tris–HCl (pH 7.6), 0.15% of Orange G, 0.03% of xylene cyanol FF, 60% glycerol and 60 mM EDTA (Fermentas, Lithuania)] and 1 μL of 100 X GelStar® (Lonza, USA). The samples were loaded into the wells, and the system was run at 75 V for 1 hour. The fragments were visualized using an Alpha Innotech® Ultraviolet Transilluminator (Alpha Innotech® USA) before the image was captured.

### PCR product purification and DNA sequencing

Wizard® SV Gel and PCR Clean-Up System (Promega, USA) were used to purify the PCR products before they were sent for sequencing. For this purpose, DNA bands of the multiplex PCR that corresponded to a particular molecular weight were sliced from high-resolution agarose gels (5%), and the purification steps were performed in accordance with the manufacturer’s instructions. DNA sequencing was outsourced to a local institution (Centre for Chemical Biology, Penang, Malaysia).

### SNP analysis

As a validation of our method, control subject nucleotide sequences were run through snpBLAST [20] and compared against the SNP database [21], where the SNPs were identified from a list with their particular SNP IDs. For example, rs1801133 for *MTHFR 677 C > T*, rs1799983 for *eNOS +894 G > T* and rs2070744 for *eNOS −786 T > C* were found.

### Genotype and allele frequencies

The expected genotype frequencies were directly calculated using the Hardy Weinberg equation (p^2^ + 2pq + q^2^ = 1).

## Results

**Figure 1:**
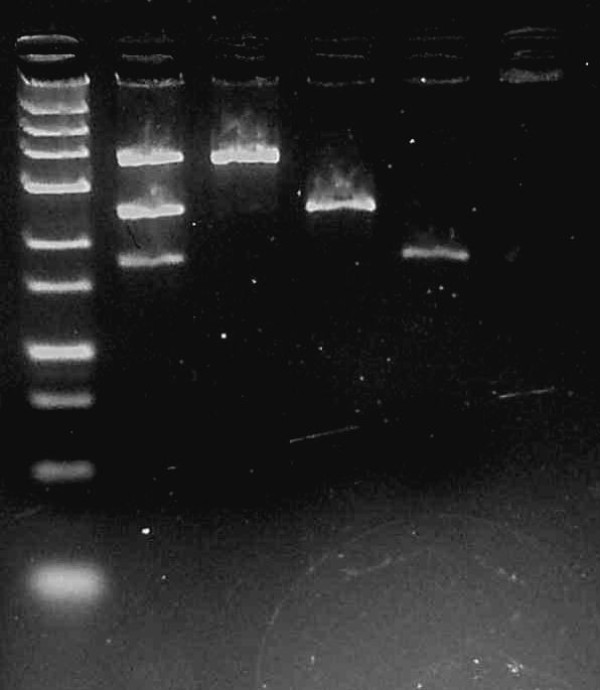
**Agarose gel with PCR products from multiplex PCR for *MTHFR 677 C > T*, *eNOS +894 G > T *and *eNOS −786 T > C*.** Lane 1 contains Fermentas O’ GeneRuler® Ultra Low Range DNA Ladder (25 bp - 700 bp); Lane 2 contains multiplex PCR products with band sizes 178 bp, 248 bp and 371 bp; Lane 3 contains a positive control for *eNOS +894 G > T* with band size 371 bp; Lane 4 contains a positive control for *MTHFR 677 C > T* with band size 248 bp; Lane 5 contains a positive control for *eNOS −786 T > C* with band size 178; and Lane 6 is a negative control.

Using this novel simultaneous detection PCR-RFLP method, a total of 114 subjects were successfully genotyped. DNA samples (n = 50) were randomly sequenced for validation of the method. As an additional confirmation, an individual with a known genotype was genotyped using a uniplex method, and the results obtained from this technique showed 100% concordance (Figure 1).

A summary of the PCR-RFLP product sizes is shown in Table 2.

**Table 2:** Summary of products and their sizes after digestion with Fermentas Fast Digest® HinfI, Fermentas Fast Digest® MspI and NEB® BanII restriction enzymes

**SNPs**	**Band size (bp)**	**Restriction enzyme**	**Restriction enzyme recognized sequences**
*MTHFR 677 C > T*		Fermentas Fast Digest® HinfI	5′-GANTC-3′
CC-Wild type	248		
CT-Heterozygote	248, 130, 118		
TT-Homozygote	130, 118		
*eNOS −786 T > C*		Fermentas Fast Digest® MspI	5′-CCGG -3′
CC-Wild type	178		
TC-Heterozygote	178, 137, 41		
TT-Homozygote	137, 41		
*eNOS +894 G > T*		NEB® BanII	5′-G(G/A)GC(T/C)C -3′
GG-Wild type	371		
GT-Heterozygote	371, 223, 139		
TT-Homozygote	223, 139		

### Amplification of *MTHFR 677 C > T* and HinfI

**Figure 2:**
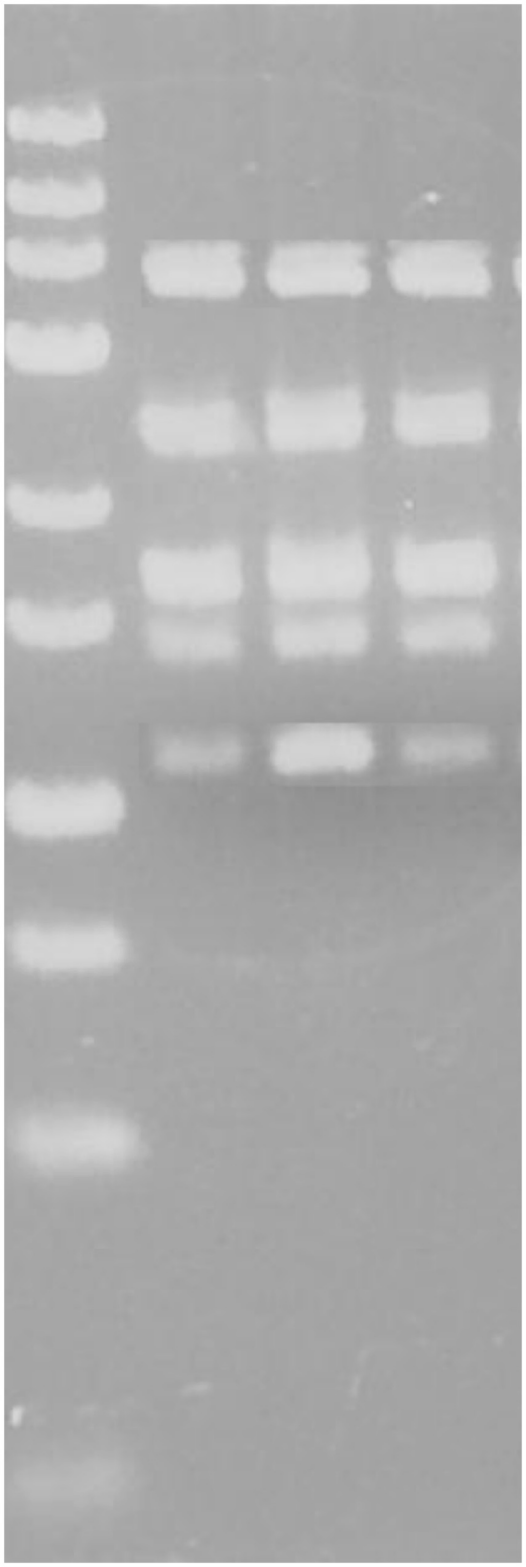
**Agarose gel with PCR–RFLP products for analysis of *MTHFR 677 C > T *and *eNOS +894 G > T *and *eNOS −786 T > C*.** Lane 1 contains Fermentas O’ GeneRuler® Ultra Low Range DNA Ladder (25 bp - 700 bp); Lanes 2, 3 and 4 contain multiplex PCR-RFLP products digested with 1 U of NEB® BanII restriction enzyme (NEB®, England), 1 U Fermentas Fast Digest® HinfI restriction enzyme (Fermentas, Lithuania) and 1 U Fermentas Fast Digest® MspI restriction enzyme (Fermentas, Lithuania), respectively. The band sizes were 371 bp, 248 bp, 178 bp, 130 bp and 118 bp for all lanes. This indicates that the sample is a TT genotype for *MTHFR 677 C > T,* GG genotype for *eNOS +894 G > T*, and TT genotype for *eNOS −786 T > C.*

The size of the PCR product for the *MTHFR 677 CT* genotype was 248 bp. The Fermentas Fast Digest® HinfI restriction enzyme recognizes the sequence 5′-GANTC-3′, and therefore, the *MTHFR 677 TT* genotype can easily be differentiated from the wild type (*MTHFR 677 CC* genotype) because two distinct bands that correspond to 118 bp and 130 bp, respectively, will be generated. In contrast, the lack of a HinfI recognition site (5′- GANCC -3′) with the C allele in *MTHFR 677 C > T* variants produces only one undigested band together with the 248 bp band. A heterozygous individual will have three bands, with two bands coming from nucleotide T and the other from nucleotide C (Figure 2). Of the 114 subjects, 50 control samples were randomly selected for DNA sequencing to identify *MTHFR 677 C > T* variants.

**Figure 3:**
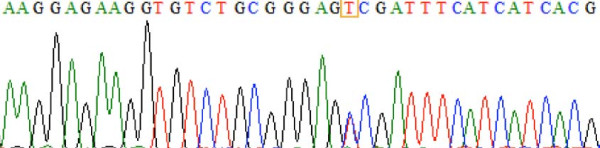
**Sequencing result confirming the *MTHFR 677 C > T *variants.** The highlighted “C” is cytosine, indicating the presence of the *MTHFR 677 C > C* variant.

**Figure 4:**
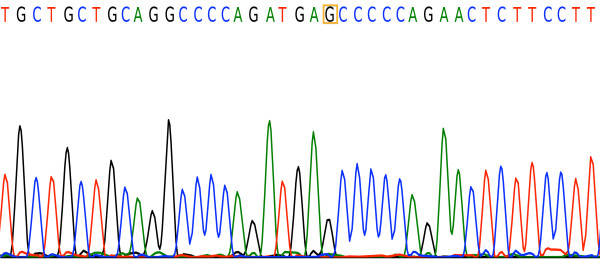
**Sequencing result confirming the *eNOS +894 G > T *variants.** The highlighted “G” is guanine, indicating the presence of the *eNOS +894 G > G* variant.

**Figure 5:**
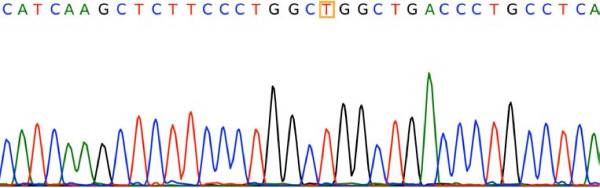
**Sequencing result confirming the *eNOS −786 C > T *variants.** The highlighted “T” is thymine, indicating the presence of the *eNOS −786 T > T* variant.

Figure 3 is a chromatogram for the sequences of 5′- GANTC -3′ tested using BioLign Alignment and Multiple PhrapEditor. The sequencing result matches that of the PCR-RFLP and snpBLAST analysis. Figures 4 and 5 are the chromatograms for the sequences of 5′-G(G/A)GC(T/C)C -3′ and 5′-CCGG -3′ respectively.

### Allele and genotype frequencies

The genotype and allele frequencies for *MTHFR 677 C > T* and *eNOS +894 G > T* and −*786 T > C* are shown in Table 3.

**Table 3:** Genotype and allele frequencies in Malaysian healthy subjects

**Polymorphisms**	**Genotype frequencies (%)**			**Allele frequencies (%)**	
*MTHFR 677 C > T*	CC	CT	TT	C	T
	0.79	0.20	0.01	0.89	0.11
*eNOS +894 G > T*	GG	GT	TT	G	T
	0.32	0.52	0.16	0.58	0.42
*eNOS −786 T > C*	CC	TC	TT	C	T
	0.05	0.15	0.80	0.13	0.87

## Discussion

Our study is the first to simultaneously determine the genotype and allele frequencies of *MTHFR 677 C > T* and *eNOS +894 G > T* and *- eNOS 786 T > C* gene polymorphisms in a Malaysian population.

We have successfully developed a new, simple method for the simultaneous detection of the *MTHFR 677 C > T* and *eNOS +894 G > T* and *eNOS −786 T > C* genetic polymorphisms. The method was successfully applied to the genotyping of 114 healthy Malay volunteers with significantly reduced pre-PCR preparation and reaction times when compared to single multiple PCR reactions.

In this study, PCR components such as the concentration of magnesium chloride, primers, dNTP and Taq DNA polymerase were optimized to achieve the lowest concentration possible while producing bands with the greatest intensity. Apart from PCR components, the PCR conditions were also optimized; generally, the lower the annealing temperature, the higher the chances of non-specific products [22].

One of the most critical steps for successful development of a new multiplex PCR method is the primer design. In this study, the primers for amplification of *MTHFR 677 C > T* were designed using Primer 3 Software, version 0.4.0 ( http://frodo.wi.mit.edu/primer3/). Both the forward and reverse *MTHFR 677 C > T* primers were under 20 base pair (bp) in length with a guanine located at the 3′ end for both forward and reverse primers to increase their specificity for the template DNA. Primer length is related to primer annealing temperature and hybridization stability [12]. An optimum primer length is between 18 and 30 bases, and the final eight to ten bases at the 3′ end playing a major role in the specificity of the primer [23].

The primer melting temperature can indicate DNA-DNA hybrid stability and is important for the optimization of PCR annealing temperature (Ta). Extremely high Ta values cause insufficient primer-template hybridization and can yield less PCR product, while low annealing temperatures can cause a high number of primer mispairings and non-specific bands [6]. The GC content is related to the primer melting temperature and plays a role in the annealing step of PCR. A previous study reported that a GC content of 45-60% results in more specific hybridization [23]. We developed our method based on the above recommendations: the *MTHFR 677 C > T* primers are 20 bp long, with 60% GC content to maintain the melting temperature above 50°C as well as to avoid the formation of non-specific bands [23].

Fast PCR and Integrated DNA Technology software was used to examine the primer melting temperature using the nearest-neighbor method [18]. More than three decades ago, Wallace et al. [19] proposed the following formula to calculate the melting temperature for primers with 18 to 30 nucleotides: (Tm, °C) = 2 (A + T) + 4 (G + C). However, this formula is applicable only to the hybridization of membrane-bound oligonucleotides and not to primer melting temperature [19]. A more recent study conducted by Allawi and SantaLucia [18] suggested the application of the nearest-neighbor method (Tm, °C = dH / (dS + R In (c/4)) $\frac{dH}{dS+R\;In\;\left(\frac{c}{4}\right)}+16.6\;\log_{10}\left[K^{+}\right]-273.15$) with the assumption of a helix-coil transition where the homoduplex DNA was considered to have a zipper formation [24].

The melting temperature of the PCR product also plays a major role in the efficiency of the multiplex PCR. The melting temperature of the PCR products can be calculated using the equation proposed by Rychlik et al. [25], where Tm = 81.5 + 0.41 x (%GC) + 16.6 log [K^+^ - (675 / length of PCR product) and [K^+^ is 50 mM.

To eliminate mispairing and the formation of any non-specific products, a hot start method was used in this study. Mispairing and primer-dimers often occur at sub-optimal primer annealing temperatures, and these mispairings can further be amplified in a PCR reaction, yielding low amounts of the specific PCR product [26]. An initial denaturation step at 95°C for 4 min is ideal, as it has been found to dissociate bound anti-Taq DNA polymerase antibody from the DNA polymerase and activates the enzyme for specific DNA amplification [26].

One unit of AmpliTaq Gold® DNA polymerase can incorporate 10 μM of dNTP per 30 min at 74°C [27]. Accordingly, 20 μM dNTP is required for the activity of 2.0 U of AmpliTaq Gold® DNA polymerase. GeneAmp® dNTP Blend was added at a concentration of 600 μM into the PCR reaction mixture because the dNTP concentration is determined by the size of the PCR amplicon. Furthermore, the unit of DNA polymerase used does not solely determine the extension time.

The annealing temperature of the PCR must ensure that both sets of forward and reverse primers bind specifically and efficiently to the template DNA. We used the formula Ta = 0.3 x Tm (primer) + 0.7 x Tm (product) -14.9 to calculate the Ta of all primers as recommended by Rychlik et al. [27]. The calculated annealing temperatures for *MTHFR 677 C > T, eNOS +894 G > T* and *eNOS −786 T > C* were 58.8°C, 62.4°C and 58.1°C, respectively. Using a gradient annealing temperature optimization, 61.1°C was found to yield three bands that corresponded to the desired SNPs. The rule of thumb for the extension step is that 1000 bases are incorporated into the DNA product every minute [28]. The extension step of the PCR was 90 seconds at 72°C, which theoretically is able to incorporate 1.5 kb of nucleotides.

To identify *MTHFR 677 C > T, eNOS +894 G > T* and *eNOS −786 T > C* variants, 1.0 U Fermentas Fast Digest® HinfI restriction enzyme, 1.0 U Fermentas Fast Digest® MspI restriction enzyme and 1.0 U of NEB® BanII restriction enzyme, respectively, were used in separate tubes to avoid errors. Restriction enzymes have the unique ability to recognize a particular DNA sequence. Therefore, a series of SNPs can be simultaneously detected from the same PCR product. To avoid double digestion of the PCR product and non-specific band formation, the PCR products should not contain the recognition site of more than one enzyme. Double digestion conditions may cause incomplete or partial digestion of PCR products, leading to interpretation as heterozygote variants instead of homozygote variants. Undoubtedly, multiple uniplex reactions have an added advantage because each gel lane represents a particular SNP as compared to multiplex reactions, where bands that correspond to other SNPs also appear in a single lane.

Polyacrylamides are hazardous to the central nervous system; thus, some researchers use non-hazardous methods such as high-resolution agarose gels (Bioline®, USA) as alternatives. With the high gel strength and small pore size, high-resolution agarose gels are thought to be comparable to polyacrylamide for discriminating small base-pair differences. In contrast to real-time PCR assays, this conventional electrophoresis system is cost effective and can be applied for routine laboratories services. Real-time PCR assays do allow for rapid SNP genotyping but use expensive Taqman approaches (probe or SYBR-green) that may not be feasible for most diagnostic laboratories.

This method was successfully used to genotype 114 healthy subjects. To validate our newly developed multiplex PCR, DNA samples were randomly selected for sequencing. In our opinion, sequencing is a more reliable method than genotyping DNA samples via conventional uniplex PCR-RFLP as conducted by Yi et al. [6] because SNPs can easily be determined by direct sequencing [29].

The sizes of the PCR products generated from our primers for *MTHFR 677 C > T*, *eNOS +894 G > T* and *eNOS −786 T > C* were 248 bp*,* 371 bp (similar to Dutra et al. [30]) and 178 bp (similar to Xin et al. [31]), respectively. A study conducted by Yi et al. [6] showed that amplified DNA sequences may be too short to be sequenced, questioning the reliability of this method. Our DNA sequencing result, however, clearly showed that genetic variants from the chromatogram were in accordance with the gel analysis, thus confirming the reliability of our new method.

The frequency of the *C* allele in healthy Malaysian Malays for *MTHFR 677 C > T* was 89%, while that for the *T* allele was 11%. This is similar to that reported by Hayati et al. [9], who reported 92.9% homozygotes and 7.1% heterozygotes. It is also comparable to previously published case–control studies of Singaporeans, where the reported allele frequencies were 82% and 18% for the C and T alleles, respectively [32]. Indians were reported to have frequencies of 83% and 17% for the C and T alleles, respectively [33]. These comparable case–control studies are in accordance with our *eNOS −786 T > C* allele frequency. However, the C allele frequency for *MTHFR 677 C > T* differed from that reported among the Chinese (30.1%) [34], Japanese (28%) [35] and Koreans (33.2%) [36]. For *eNOS −786 T > C,* the C allele frequency differed from that of the Iranians (21.7%) [37] and the Southern Americans (5%) [38]. These differences may be due to differences in genetic make-up among the different ethnic populations.

The G and T allele frequencies for *eNOS +894 G > T* were 0.58 and 0.42, respectively, which is similar to that reported for France [39], the United Kingdom [40] and Tunisia [41]. However, this allele frequency was different from previously published reports on Malaysian Malays [10], Singaporeans [32] and Chinese [2], while the reported T allele frequency for these three populations, particularly the Chinese, was as low as 0.11 [2]. The discrepancy in allele frequency between our study and that of Ghazali et al. [10] may be due to the different populations and sampling. In our study, the healthy Malay subjects were randomly selected from students and staff from our institution, while in their study, the control subjects were age- and sex matched to the hypertensive patients; they were thus not randomly selected.

## Conclusions

The newly developed simultaneous detection method for the three SNPs is simple, inexpensive and reproducible and can be routinely applied in most laboratories for the identification of allelic and genotypic frequencies.

## Competing interests

The authors declare that they have no competing interests.

## Authors' contributions

LKW collected clinical samples, designed the primers, performed and optimized the multiplex PCR-RFLP, analyzed clinical samples and drafted the manuscript. LRG contributed to initial study design, protocol development and manuscript revision. GSH contributed to initial study design, protocol development and manuscript revision and gave the final approval for publishing the manuscript as the team leader. All authors read and approved the final manuscript.

## Pre-publication history

The pre-publication history for this paper can be accessed here:

http://www.biomedcentral.com/1471-2350/13/34/prepub

## References

[B1] Maitland-Van DZAH Lynch A Boerwinkle E Arnett DK Davis BR Leiendecker-Foster C Ford CE Eckfeldt JH Interactions between the single nucleotide polymorphisms in the homocysteine pathway (MTHFR 677 C > T, MTHFR 1298 A > C, and CBSins) and the efficacy of HMG-CoA reductase inhibitors in preventing cardiovascular disease in high-risk patients of hypertension: the GenHAT study Pharmacogenet Genomics 2008 18 651 656 10.1097/FPC.0b013e3282fe1759 18622257 PMC2729516

[B2] Cheng J Liu J Li X Yu L Peng J Zhang R Geng Y Nie S Effect of polymorphisms of endothelial nitric oxide synthase on ischemic stroke: a case– control study in a Chinese population Clinica Chimica Acta 2008 392 46 51 10.1016/j.cca.2008.03.004 18396156

[B3] Scher AI Terwindt GM Verschuren WMM Kruit MC Blom HJ Kowa H Frants RR van den Maagdenberg AMJM van Buchem M Ferrari MD Launer LJ Migraine and MTHFR C677T genotype in a population-based sample Ann Neurol 2006 59 372 375 10.1002/ana.20755 16365871

[B4] Lim JS Kwon HM Risk of silent “stroke” in patients older than 60 years: risk assessment and clinical perspective Clin Interv Aging 2010 5 239 251 20852671 10.2147/cia.s7382 PMC2938031

[B5] Barbaux S Kluijtmans LA Whitehead AS Accurate and rapid “multiplex heteroduplexing” method for genotyping key enzymes involved in folate/homocysteine metabolism Clin Chem 2000 46 907 912 10894832

[B6] Yi P Pogribny IP Jill James S: Multiplex PCR for simultaneous detection of 677 C > T and 1298 A > C polymorphisms in methylenetetrahydrofolate reductase gene for population studies of cancer risk Cancer lett 2002 181 209 213 10.1016/S0304-3835(02)00060-5 12175537

[B7] Koksal V Baris I Etlik O Primer-engineered multiplex PCR-RFLP for detection of MTHFR C677T, prothrombin G20210A and factor V Leiden mutations Exp Mol Pathol 2007 83 1 3 10.1016/j.yexmp.2006.12.006 17275807

[B8] Agarwal RP Peters SM Shemirani M Von Ahsen N Improved real-time multiplex polymerase chain reaction detection of methylenetetrahydrofolate reductase (MTHFR) 677 C > T and 1298A > C polymorphisms using nearest neighbour model-based probe design J Mol Diagn 2007 9 345 350 10.2353/jmoldx.2007.060035 17591934 PMC1899423

[B9] Hayati AR Zainal AI Tan GC Ong LC Khoo TB MTHFR C677T polymorphism as a risk factor of neural tube defects in Malay: a case control study Med J Malaysia 2008 63 379 383 19803295

[B10] Ghazali DM Rehman A Rahman ARA Candidate gene polymorphisms and their association with hypertension in Malays Clin Chim Acta 2008 388 46 50 10.1016/j.cca.2007.10.002 17977523

[B11] Loo KW Gan SH Burden of stroke in Malaysia Int J Stroke 2012 7 165 167 10.1111/j.1747-4949.2011.00767.x 22264370

[B12] Qiagen® Multiplex PCR Handbook 2010 [ www.qiagen.com/hb/multiplexpcr]

[B13] Santella RM Approaches to DNA/RNA extraction and whole genome amplification Cancer Epidemiol Biomarkers Prev 2006 15 1585 1587 10.1158/1055-9965.EPI-06-0631 16985017

[B14] The Primer 3 Software version 0.4.0 [ http://frodo.wi.mit.edu/primer3/]

[B15] The Basic Local Alignment Search Tool (BLAST) Database [ http://blast.ncbi.nlm.nih.gov/Blast.cgi]

[B16] The UCSC Genome Bioinformatics Database

[B17] The Restriction Mapper version 3 [ http://www.restrictionmapper.org/]

[B18] Dutra AV Lin HF Juo SHH Boyadjis M Moussouttas M Reddy PL Grewal RP Analysis of the endothelial nitric oxide synthase gene as a modifier of the cerebral response to ischemia J Stroke Cerebrovasc Dis 2006 15 128 131 10.1016/j.jstrokecerebrovasdis.2006.03.002 17904064

[B19] Xin Y Song X Xue H Liu Z Wang X Wang H Sun K Bai Y Liu J Hui R A common variant of the eNOS gene (E298D) is an independent risk factor for left ventricular hypertrophy in human essential hypertension Clin Sci 2009 117 67 73 10.1042/CS20080476 19132956

[B20] The Single Nucleotide Polymorphisms BLAST (snp-BLAST) Database [ http://www.ncbi.nlm.nih.gov/SNP/snpblastByChr.html]

[B21] The Single Nucleotide Polymorphisms (SNP) Database [ http://www.ncbi.nlm.nih.gov/snp]

[B22] Burpo FJ A critical review of PCR primer design algorithms and cross hybridization case study Biochemistry 2001 218 1 12

[B23] Abd-Elsalam K Minireview-Bioinformatic tools and guideline for PCR primer design Afr J Biotechnol 2003 2 91 95

[B24] Allawi HT SantaLucia J Thermodynamics and NMR of internal G.T mismatches in DNA Biochemistry 1997 36 10581 10594 10.1021/bi962590c 9265640

[B25] Wallace RB Shaffer J Murphy RF Bonner J Hirose T Itakura K Hybridization of synthetic oligodeoxyribonucleotides to phi chi 174 DNA: the effect of single base pair mismatch Nucleic Acids Res 1979 6 3543 3557 10.1093/nar/6.11.3543 158748 PMC327955

[B26] Le Novere N MELTING, computing the melting temperature of nucleic acid duplex Bioinformatics 2001 17 1226 1227 10.1093/bioinformatics/17.12.1226 11751232

[B27] Rychlik W Spencer WJ Rhoads RE Optimization of the annealing temperature for DNA amplification in vitro Nucleic Acids Res 1990 18 6409 6412 10.1093/nar/18.21.6409 2243783 PMC332522

[B28] Birch DE Kolmodin L Wong J Zangenberg GA Zoccoli MA McKinney N Young KKY Simplified hot start PCR Nature 1996 381 445 446 10.1038/381445a0 8632804

[B29] Innis MA Myambo KB Gelfand DH Brow MA DNA sequencing with Thermus aquaticus DNA polymerase and direct sequencing of polymerase chain reaction-amplified DNA Proc Natl Acad Sci 1988 85 9436 9440 10.1073/pnas.85.24.9436 3200828 PMC282767

[B30] Delidow BC Molecular cloning of PCR fragments with cohesive ends Mol Biotechnol 1997 8 53 60 10.1007/BF02762339 9327397

[B31] Kwok PY Duan S SNP discovery by direct DNA sequencing Methods Mol Biol 2003 212 71 84 12491904 10.1385/1-59259-327-5:071

[B32] Moe KT Woon FP De Silva DA Wong P Koh TH Kingwell B Chin-Dusting J Wong MC Association of acute ischemic stroke with the MTHFR C677T polymorphism but not with NOS3 gene polymorphisms in a Singapore population Eur J Neurol 2008 15 1309 1314 10.1111/j.1468-1331.2008.02308.x 19049547

[B33] Somarajan BI Kalita J Mittal B Misra UK Evaluation of MTHFR C677T polymorphism in ischemic and hemorrhagic stroke patients. A case– control study in a Northern Indian population J Neurol Sci 2011 304 67 70 10.1016/j.jns.2011.02.010 21406306

[B34] Feng LG Song ZW Xin F Hu J Association of plasma homocysteine and methylenetetrahydrofolate reductase C677T gene variant with schizophrenia: a Chinese Han population-based case–control study Psychiatry Res 2009 168 205 208 10.1016/j.psychres.2008.05.009 19564051

[B35] Kawamoto R Kohara K Oka Y Tomita H Tabara Y Miki T An association of 5,10-methylenetetrahydrofolate reductase (MTHFR) gene polymorphism and ischemic stroke J Stroke Cerebrovasc Dis 2005 14 67 74 10.1016/j.jstrokecerebrovasdis.2004.12.003 17904003

[B36] Moon HW Kim TY Oh BR Min HC Cho HI Bang SM Lee JH Yoon SS Lee DS MTHFR 677CC/1298CC genotypes are highly associated with chronic myelogenous leukemia: a case–control study in Korea Leuk Res 2007 31 1213 1217 10.1016/j.leukres.2006.10.016 17156840

[B37] Safarinejad MR Khoshdel A Shekarchi B Taghva A Safarinejad S Association of the T-786 C, G894T and 4a/4b polymorphisms of the endothelial nitric oxide synthase gene with vasculogenic erectile dysfunction in Iranian subjects BJU Int 2011 107 1994 2011 10.1111/j.1464-410X.2010.09755.x 20955262

[B38] Khurana VG Sohni YR Mangrum WI McClelland RL O’Kane DJ Meyer FB Meissner I Endothelial nitric oxide synthase T-786 C single nucleotide polymorphism: a putative genetic marker differentiating small versus large ruptured intracranial aneurysms Stroke 2003 34 2555 2559 10.1161/01.STR.0000096994.53810.59 14576373

[B39] Elbaz A Poirier O Moulin T Chedru F Cambien F Amarenco P Association between the Glu298Asp polymorphism in the endothelial constitutive nitric oxide synthase gene and brain infarction Stroke 2000 31 1634 1639 10.1161/01.STR.31.7.1634 10884465

[B40] Hassan A Gormley K O’Sullivan M Knight J Sham P Vallance P Bamford J Markus H Endothelial nitric oxide gene haplotypes and risk of cerebral small-vessel disease Stroke 2004 35 654 659 10.1161/01.STR.0000117238.75736.53 14963277

[B41] Ezzidi I Mtiraoui N Mohamed MB Mahjoub T Kacem M Almawi WY Association of endothelial nitric oxide synthase Glu298Asp, 4b/a, and -786 T > C gene variants with diabetic nephropathy J Diab Complications 2008 22 331 338 10.1016/j.jdiacomp.2007.11.011 18413207

